# Biosynthetic approaches to efficient assimilation of CO_2_ *via* photorespiration modification in plant chassis

**DOI:** 10.3389/fbioe.2022.979627

**Published:** 2022-08-08

**Authors:** Qing Wang, Hao Yang, Peijian Cao, Fangjian Chen, Lei Zhao

**Affiliations:** ^1^ Key Laboratory of Systems Microbial Biotechnology, Tianjin Institute of Industrial Biotechnology, Chinese Academy of Sciences, Tianjin, China; ^2^ National Center of Technology Innovation for Synthetic Biology, Tianjin, China; ^3^ College of Biotechnology, Tianjin University of Science and Technology, Tianjin, China; ^4^ China Tobacco Gene Research Center, Zhengzhou Tobacco Research Institute of CNTC, Zhengzhou, China

**Keywords:** carbon fixation, plant chassis, metabolic pathway design, photorespiration, bioproduction

## Abstract

Plant chassis has emerged as the platform with great potential for bioproduction of high value-added products such as recombinant protein, vaccine and natural product. However, as the primary metabolic pathway, photorespiration results in the loss of photosynthetically fixed carbon compounds and limits the exploration of plant chassis. People are endeavored to reduce the photorespiration energy or carbon loss based on variation screening or genetic engineering. Insomuch as protein engineering of Rubisco has not resulted in the significant improvement of Rubisco specificity which is linked to the direct CO_2_ fixation, the biosynthetic approaches of photorespiration bypass are gaining much more attention and manifested great potentiality in conferring efficient assimilation of CO_2_ in plant chassis. In this review, we summarize the recent studies on the metabolic pathway design and implementation of photorespiration alternative pathway aiming to provide clues to efficiently enhance carbon fixation *via* the modification of photorespiration in plant chassis for bioproduction. These will benefit the development of plant synthetic metabolism for biorefineries *via* improvement of artificial carbon sequestration cycle, particularly for the mitigation of serious challenges such as extreme climate change, food and energy shortages in the future.

## Introduction

As the global human population rapidly increases, new and efficient biological systems are urgent to be obtained to meet the growing demand for resources (Rai et al., 2019). Synthetic biology has been established to be one of the powerful platforms that focuses on the design of novel synthetic biological pathways or redesign of existing natural systems which could fulfill the requirement mentioned above (Holland and Jez, 2018). Although microbial chassises are widely used in present industrial bioproduction, their improvement is greatly challenged due to the lack of post-translational modifications, compartmentalization, non-functional nature and negligible activity of some proteins (Tiwari et al., 2021). Plant chassis, gradually emerging as the platform with great potential for bioproduction of high value-added products through manipulation of synthetic biology, is becoming the ideal and sustainable platform for their ability to directly use sunlight and CO_2_ to generate a variety of organic compounds (Fesenko and Edwards, 2014). Thus, plant synthetic biology is expected to present great potentiality in leading the development of molecular farming to benefit the production of food, fuels, fodder, therapeutics and environmental welfare to create totally synthetic life forms or components (Rai et al., 2019; Fausther-Bovendo and Kobinger, 2021; Cournoyer et al., 2022).

In optimizing the plant chassis which is suitable for the bioproduction of value-added metabolites, people are mostly focusing on the modification of carbon flux in C_3_ plants such as tobacco, rice and tomato. Ribulose-1,5-bisphosphate carboxylase oxygenase (Rubisco) is the first and important enzyme in C_3_ pathway to fix atmospheric CO_2_ (Sage et al., 2012). Both 3-PGA and 2-PG are generated due to the enzymatic activity of Rubisco (Lorimer, 1981; Betti et al., 2016). 2-PG, which could not be directly used for carbon fixation, inhibits the activity of chloroplastic enzymes (Anderson, 1971; Norman and Colman, 1991). To degrade 2-PG, a photorespiration pathway was developed to recycle 2-PG into 3-PGA that re-enter the Calvin-Benson cycle during evolution, through a serial of catalysis in chloroplast, peroxisome, mitochondrion and cytosol (Bauwe et al., 2010). During the process, two molecules of 2-PG are converted into one molecule of 3-PGA and one carbon atom is lost as CO_2_ in the mitochondria (Peterhansel et al., 2010), resulting in 25% of carbon loss (Walker et al., 2016). Furthermore, the photorespiratory effects could be enhanced by serious conditions such as high temperature and water shortage (Sharkey, 1988; Walker et al., 2016). Intensive studies are tentatively performed to decrease the carbon loss in photorespiration by genetic manipulation of Rubisco aiming to improve its selectivity and kinetic properties but without great effects (Bathellier et al., 2018; South et al., 2018). By contrast, the design of novel photorespiratory bypass by biosynthetic approaches has brought attention and is thought to play a major role in reducing carbon release of native photorespiration. To date, several novel photorespiratory bypasses have been implemented into plants and remarkably developed (Kebeish et al., 2007; Carvalho et al., 2011; South et al., 2019; Shen et al., 2019; Roell et al., 2021). The present review summarizes the novel biosynthetic pathways of reducing carbon release *via* the design of photorespiratory bypasses, and analyzes their potential effects. Then future perspective is suggested aiming to provide people with enlightenment to make progressive development in this field.

## Biosynthetic photorespiratory bypasses implemented into plants

**FIGURE 1:**
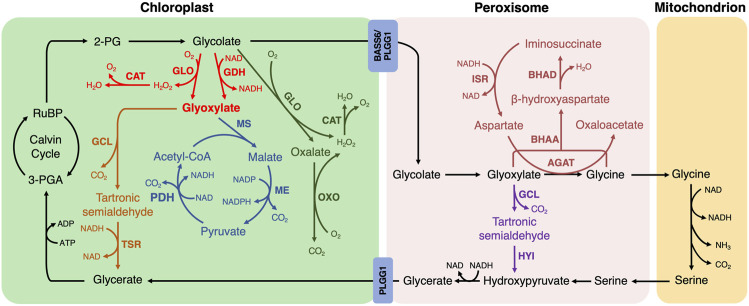
The photorespiratory bypasses implemented into plants. The natural photorespiratory pathway and biosynthetic bypasses to photorespiration are marked by defined color. BASS, Bile acid sodium symporter; PLGG, glycolate-glycerate transporter. The first bypass in the chloroplast (red and orange; Kebeish et al., 2007; Dalal et al., 2015; South et al., 2019; Chen et al., 2019; Wang et al., 2020; Nayak et al., 2022; Zhang et al., 2022): GLO, glycolate oxidase; CAT, catalase; GDH, glycolate dehydrogenase; GCL, glyoxylate carboligase; TSR, tartronic semialdehyde reductase. The second bypass in the chloroplast (red and blue; Maier et al., 2012; South et al., 2019; Cavanagh et al., 2022): MS, malate synthase; ME, malic enzyme; PDH, pyruvate dehydrogenase. The third bypass in the chloroplast (green; Shen et al., 2019): OXO, oxalate oxidase. The first bypass in the peroxisome (purple; Carvalho et al., 2011): HYI, hydroxypyruvate isomerase. The second bypass in the peroxisome (dark red; Roell et al., 2021): AGAT, aspartate:glyoxylate aminotransferase; BHAA, β-hydroxyaspartate aldolase; BHAD, β-hydroxyaspartate dehydratase; ISR, iminosuccinate reductase.

1. Among the reported photorespiratory bypass pathways implemented into plants, *E. coli-*originated glyoxylate oxidation catalysis has been extensively tested (Kebeish et al., 2007; Dalal et al., 2015; South et al., 2019; Chen et al., 2019; Wang et al., 2019; Nayak et al., 2022; Zhang et al., 2022). Glycolate is converted into glyoxylate by glycolate dehydrogenase (GDH) or glycolate oxidase (GLO), followed by the generation of tartronic semialdehyde and CO_2_ from two molecules of glyoxylate catalyzed by glyoxylate carboligase (GCL). Tartronic semialdehyde is then converted to glycerate by tartronic semialdehyde reductase (TSR) in the chloroplast, and all the catalytic steps are established in the chloroplast (Figure 1, fonts marked by red and orange color). H_2_O_2_, as the by-product of GLO-mediated catalysis, is decomposed *via* the introduction of catalase (CAT) (Wang et al., 2019).

This bypass manifested several advantages compared to the natural photorespiration pathway. Firstly, CO_2_ is shifted and released into the chloroplast, where it could be re-cycled into Calvin-Bension metabolism. Secondly, the NH_3_ production is greatly avoided. Thirdly, the metabolite transport between organelles is bypassed, whereas between 14 and 18 transport processes are required in natural photorespiration (Reumann and Weber, 2006). Therefore, it saves more energy than the native photorespiratory. Expectedly, the reduced photorespiration, improved photosynthetic performance and enhanced biomass production were observed in *Arabidopsis*, camelina and cucumber (Kebeish et al., 2007; Dalal et al., 2015; Chen et al., 2019). In detail, CO_2_ compensation point was significantly decreased in transgenic *Arabidopsis* with a reduction of more than 10% (Kebeish et al., 2007). Transgenic camelina showed 14–28% increase in CO_2_ fixation (Dalal et al., 2015). The same effects on biomass and grain yield were obtained when it was introduced into rice (Wang et al., 2019; Nayak et al., 2022), however, the seeding rate is decreased and chalky rice rate is increased, which could be explained by the undelivered photosynthetic carbohydrates into grains in a timely or efficient way during the filling stage (Wang et al., 2019; Zhang et al., 2022).

2. In the second bypass, a cycle to completely decarboxylate glycolate is introduced into the chloroplast (Maier et al., 2012; South et al., 2019). Glycolate is oxidized to glyoxylate by GDH or GLO, which is the same as *E. coli* glyoxylate oxidation bypass. Glyoxylate and acetyl-CoA are then converted to malate using malate synthase (MS), and the resulting malate is decarboxylated into pyruvate by the malic enzyme (ME) with the first CO_2_ release. Pyruvate is oxidized into acetyl-CoA by pyruvate dehydrogenase (PDH) with the second CO_2_ release. The acetyl-CoA re-enters into the bypass by combining with glyoxylate (Figure 1, fonts marked by red and blue color).

This bypass shifts CO_2_ release to the chloroplast with no NH_3_ production as in the first bypass, but impedes the Calvin-Benson cycle due to no 3-PGA recovery (Peterhansel et al., 2013). It has already been successfully implemented into *Arabidopsis* and tobacco (Maier et al., 2012; South et al., 2019). The *Arabidopsis* transgenic lines exhibited various phenotypes of leaf color and oxidative lesions possibly due to their variation of CAT activity in chloroplasts, which remains to be explained (Maier et al., 2012). CO_2_ compensation point was not statistically different between genotypes in *Arabidopsis* (Maier et al., 2012)*.* Consistent with the results of *Arabidopsis,* 24% of the transgenic tobacco with *GLO* and *CAT* presented stunted growth and yellow leaves. However, the transgenic tobacco, in which GDH is used instead of GLO to remove the need for CAT, showed a biomass increase of 18% and 24% and a CO_2_ compensation point decrease of 6.4% and 10% under wild type and *PLGG1* RNAi background when compared with control, respectively (South et al., 2019). Meanwhile, the transgenic tobacco line with *PLGG1* RNAi module sustained 19% less yield loss compared to wild type under high temperature conditions (Cavanagh et al., 2022). These results suggest that the introduction of GDH into plants without producing H_2_O_2_ is a valuable strategy to improve the agronomic trait or phenotype.

3. In the third bypass, glycolate is converted to CO_2_ completely by endogenous enzymes in the chloroplast (Shen et al., 2019). Glycolate is oxidized to oxalate and H_2_O_2_ by GLO in two steps, and oxalate is then oxidized to CO_2_ and H_2_O_2_ by oxalate oxidase (OXO). The by-product H_2_O_2_ is also scavenged by CAT (Figure 1, fonts marked by green color). The chloroplastic CO_2_ concentration is enhanced and NH_3_ release is bypassed in this design. Nevertheless, no additional reducing equivalent is generated and more ATP units are required (Shen et al., 2019). When the bypass is introduced into rice, CO_2_ compensation point is decreased and biomass production is enhanced but grain yield varied by sowing seasons and setting rate was decreased (Shen et al., 2019). In addition, the decreased head milled rice rate and increased chalky rice rate were observed in the transgenic rice lines, indicating that milling quality and appearance quality was reduced to some extent (Zhang et al., 2022). Furthermore, this bypass and the first bypass using the rice-self originating CAT in transgenic rice did not show oxidative damage compared to the second bypass using *E. coli* sourced CAT in transgenic *Arabidopsis* and tobacco (Maier et al., 2012; Shen et al., 2019; South et al., 2019; Wang et al., 2019), suggesting that endogenous enzyme may more efficient to scavenge H_2_O_2_.

4. The first bypass located in the peroxisome is a simplified version of *E. coli* glyoxylate oxidation catalysis (Carvalho et al., 2011). The decarboxylation reaction of glyoxylate is catalyzed by the same enzyme GCL. Tartronic semialdehyde is then converted to hydroxypyruvate fed back into photorespiration by hydroxypyruvate isomerase (HYI) (Figure 1, fonts marked by purple color). NH_3_ production is avoided but only three-quarters of carbon is converted to 3-PGA (Eisenhut et al., 2008). Since HYI protein was not detected in transgenic tobacco, this bypass has been partially established in tobacco (Carvalho et al., 2011). Leaves of transgenic tobacco exhibited growth retardation and lesions after exposure to ambient air but not at an increased concentration of CO_2_, indicating the presence of a metabolic defect associated with photorespiration nitrogen cycle. These results suggest that the metabolic flux through glyoxylate to tartronic semialdehyde directly or indirectly caused deleterious effects on plants and the impact of this bypass remains to be proven (Carvalho et al., 2011).

5. Another bypass located in the peroxisome is a β-hydroxyaspartate cycle also starts from glyoxylate (Schada von Borzyskowski et al., 2019). Glyoxylate and aspartate are first converted into glycine and oxaloacetate by aspartate:glyoxylate aminotransferase (AGAT). Then the resulting glycine and glyoxylate are condensed into β-hydroxyaspartate using β-hydroxyaspartate aldolase (BHAA). β-hydroxyaspartate is converted to iminosuccinate in the presence of β-hydroxyaspartate dehydratase (BHAD). Iminosuccinate is reduced to aspartate by iminosuccinate reductase (ISR) to regenerate the amino group donor was regenerated for the first step of this bypass (Figure 1, fonts marked by dark red color). Oxaloacetate, generated in this bypass, could directly enter the tricarboxylic acid cycle or be used as the substrate for anabolic reactions without carbon and nitrogen loss (Schada von Borzyskowski et al., 2019; Roell et al., 2021). Roell et al. (2021) further investigated this bypass by using *Arabidopsis ggt1-1* mutant in which glutamate glyoxylate aminotransferase 1 is down-regulated, thereby directing metabolite flux toward this biosynthetic bypass. The transgenic plants under mutant background were increased in growth but did not significantly affect the CO_2_ compensation point compared with *ggt1-1* mutant in ambient air (Roell et al., 2021). However, due to the multiple effects of oxaloacetate metabolism such as tricarboxylic acid cycle and amino acid biosynthesis, the full potential of this bypass may be masked (Roell et al., 2021).

## Potentially achieved photorespiratory bypasses in plants

**FIGURE 2:**
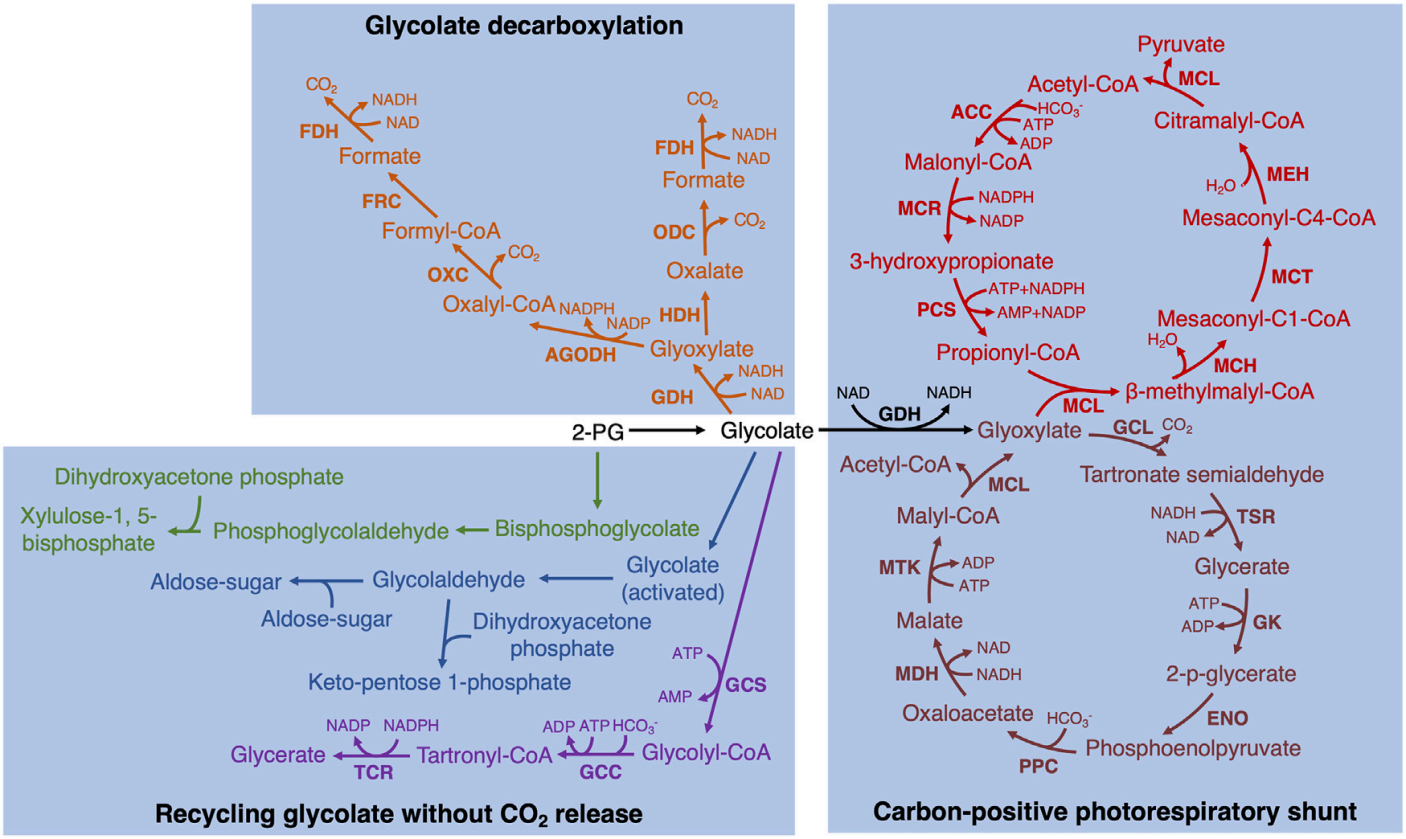
The potentially achieved biosynthetic bypasses of photorespiration in plants. Two glycolate decarboxylation bypasses are marked by orange (Eisenhut et al., 2008; Claassens et al., 2020; Khurshid et al., 2020). GDH, glycolate dehydrogenase; HDH, hydroxyacid dehydrogenase; ODC, oxalate decarboxylase; FDH, formate dehydrogenase; AGODH, CoA-acylating glyoxylate dehydrogenase; OXC, oxalyl-CoA decarboxylase; FRC, formyl-CoA transferase. Two different hypothetical bypasses to recycle glycolate without CO_2_ release are marked by green (Ort et al., 2015) and blue (Bar-Even, 2018), respectively. Tartronyl-CoA pathway (purple; Scheffen et al., 2021): GCS, glycolyl-CoA synthetase; GCC, glycolyl-CoA carboxylase; TCR, tartronyl-CoA reductase. The bypass converting glycolate to pyruvate (black and red; Shih et al., 2014): MCL, malyl-CoA lyase; MCH, mesaconyl-C1-CoA hydratase; MCT, mesaconyl-CoA C1:C4 CoA transferase; MEH, mesaconyl-C4-CoA hydratase; ACC, acetyl-CoA carboxylase; MCR, malonyl-CoA reductase; PCS, propionyl-CoA synthase. The bypass converting glycolate to acetyl-CoA (black and dark red; Yu et al., 2018): GCL, glyoxylate carboligase; TSR, tartronic semialdehyde reductase; GK, glycerate kinase; ENO, enolase; PPC, phosphoenolpyruvate carboxylase; MDH, malate dehydrogenase; MTK, malate thiokinase.

In addition to the implemented bypasses described above, there are some promising alternative bypasses that could be experimentally tested in plants. Such as similar to the third bypass in the chloroplast, glycolate is converted to two molecules of CO_2_ completely (Eisenhut et al., 2008; Claassens et al., 2020; Khurshid et al., 2020). In these bypasses, glycolate is first converted to formate with one molecule of CO_2_ release by three or four enzymes and then formate oxidizes to CO_2_ by formate dehydrogenase (FDH) (Eisenhut et al., 2008; Claassens et al., 2020; Khurshid et al., 2020) (Figure 2, fonts marked by orange color). In addition, it is also possible to design new bypasses. For example, glyoxylate can spontaneously convert to formate and CO_2_ in the presence of H_2_O_2_ (Wingler et al., 1999). Therefore, GLO and FDH could be combined with the above non-enzymatic reaction together to completely decarboxylate glycolate into two molecules of CO_2_.

Furthermore, the carbon release is always detected in photorespiration and alternative pathways need to be tested in plants that could recycle glycolate without CO_2_ release. One hypothetical bypass is to reduce 2-PG to phosphoglycolaldehyde, which is then combined with dihydroxyacetone phosphate to produce xylulose bisphosphate. Then xylulose bisphosphate can be dephosphorylated to xylulose-5-phosphate back into Calvin-Benson cycle (Ort et al., 2015) (Figure 2, fonts marked by green color). Another hypothetical carbon re-cycle route is to reduce glycolate to glycolaldehyde *via* a glycolyl-phosphate or glycolyl-CoA intermediate, and then glycolaldehyde is as an acceptor or donor by an aldol reaction into the Calvin-Benson cycle (Bar-Even, 2018) (Figure 2, fonts marked by blue color). In addition, Trudeau et al. (2018) utilized the natural and artificially designed enzymes to identify some synthetic carbon-conserving bypasses and provide principles of the alternative biosynthesis. Recently, by applying rational design and high-throughput evolution of enzymes, one of the bypasses, tartronyl-CoA pathway, has been successfully reconstituted and implemented *in vitro* (Scheffen et al., 2021) (Figure 2, fonts marked by purple color). This bypass could directly convert glycolate to glycerate by only three enzymes and fix an additional carbon, increasing the carbon efficiency from 75% to 150%. The other approach is to engineer bypass involving intermediates not present in plants or design a single enzyme to convert glycolate to glycerate directly in the chloroplast.

A carbon-positive photorespiratory shunt for converting downstream products was suggested as a strategy beyond zero-carbon release bypasses. A promising alternative pathway that needs to be tested in plants is to convert glycolate to pyruvate, which requires introducing seven enzymes and fixing one HCO_3_^−^ (Shih et al., 2014). This bypass prevented the loss of NH_4_^+^ while increasing the carbon fixation rate (Figure 2, fonts marked by black and red color). In addition, Yu et al. (2018) designed a synthetic malyl-CoA-glycerate pathway to assimilate glycolate to produce acetyl-CoA by releasing one molecule of CO_2_ and fixing one molecule of HCO_3_^−^ to achieve no carbon loss (Figure 2, fonts marked by black and dark red color).

## Perspective on the modification of photorespiration by synthetic biology

The reported bypasses may be limited by contingencies of evolutionary change and natural selection due to the attempts to reduce carbon release are inferred from biochemistry and theory (Peterhansel et al., 2013b). The substrate affinity, optimal pH and sensitivity of inhibitors need to be adjusted to support high fluxes in novel pathways considering that the introduced enzymes are not active enough when implemented into plant cells (Rademacher et al., 2002; Peterhansel et al., 2013b). In addition, since most of the bypasses are established *in vitro*, it is necessary to clone entire biosynthetic bypasses gradually into a single construct for transformation of a single plant to better assess these alternative approaches. Multiple designs need to be tested for combinations of promoter genes to optimize gene expression (South et al., 2018). Furthermore, it is possible to turn off the native photorespiratory pathway by using mutants or gene knockout approaches to maximize flux for testing alternative pathways (South et al., 2019; Roell et al., 2021).

Since most of the bypasses are targeted to the chloroplast, it is necessary to have efficient and precise chloroplast transit peptides (CTP) to target enzymes into the chloroplast. CTP recognition is governed by sequence-independent interactions and vectorial-specific recognition domains by a series of *in vitro* and *in vivo* experiments (Chotewutmontri et al., 2012). Shen et al. (2017) revealed that a 21 amino acid unfolded region in the N-terminus of CTP is important to efficiently import proteins into the chloroplast. In addition, Wimmer et al. (2017) found the specific but not conserved sequence elements of CTP could target proteins to the peripheral chloroplasts rather than central chloroplasts in a single-cell C_4_ plant. These results suggest that the optimization of CTP sequence is crucial to effectively target diverse enzymes into the chloroplasts.

The availability of both reducing equivalent and ATP is crucial to bioproduction. In plants, ATP and NADPH are generated in the process of photosynthesis in the chloroplast (Voon and Lim, 2019), and NADH is mainly generated in the mitochondria by the tricarboxylic acid cycle (Maynard and Kanarek, 2020). However, since most dehydrogenases function with NADH as a cofactor, the sufficient supply of NADH is essential for the production of dehydrogenase-derived chemicals (Meng et al., 2021). Therefore, ferredoxin-NAD^+^ reductase could be introduced into the chloroplast to generate NADH for increasing reducing equivalents. More photorespiratory bypasses that depend on NADH can also be achieved in the chloroplast in the future.

The pathway targeted to the mitochondrion has not been evaluated for the establishment of photorespiratory bypasses. Glycine and serine from photorespiration could serve as the substrates for bypass design in the mitochondrion. Glycine is important for synthesis of collagen, elastin and other protein in mammals (Meléndez-Hevia and de Paz-Lugo, 2008). Perhaps the new bypass introduction might convert glycine to collagen to reduce carbon release and increase target product. Serine could be converted to pyruvate (Yu and Liao, 2018) or cysteine (Busch, 2020) to participate in the tricarboxylic acid cycle or sulfate assimilation, respectively. However, the bypasses using serine as a substrate could only reduce the downstream reactions of photorespiration, but cannot reduce the CO_2_ and NH_3_ release.

In conclusion, novel and technological solutions must be obtained to further increase the productivity of crops because traditional genetic engineering may reach a plateau (Batista-Silva et al., 2020). Synthetic biology is opening up a new opportunity, that is, possible to conceptualize, design, build and test multiple approaches to redesign photorespiration to improve plant growth and yield (South et al., 2018). However, more fundamental researches and more advanced biosynthetic approaches are needed to effectively reduce carbon release in plant chassis. It is necessary to assess the pathways not only in model plants but also in food crops under a range of relevant agricultural environments to benefit the development of plant synthetic metabolism.
